# Immunopathogenicity of *Acanthamoeba* spp. in the Brain and Lungs

**DOI:** 10.3390/ijms22031261

**Published:** 2021-01-27

**Authors:** Karolina Kot, Natalia Łanocha-Arendarczyk, Danuta Kosik-Bogacka

**Affiliations:** 1 Department of Biology and Medical Parasitology, Faculty of Pharmacy, Medical Biotechnology and Laboratory Medicine, Pomeranian Medical University in Szczecin, Powstańców Wielkopolskich 72, 70-111 Szczecin, Poland; kotkar@pum.edu.pl (K.K.); nlanocha@pum.edu.pl (N.Ł.-A.); 2 Independent Laboratory of Pharmaceutical Botany, Faculty of Pharmacy, Medical Biotechnology and Laboratory Medicine, Pomeranian Medical University in Szczecin, Powstańców Wielkopolskich 72, 70-111 Szczecin, Poland

**Keywords:** *Acanthamoeba* spp., brain, lungs, molecular mechanisms

## Abstract

Free-living amoebas, including *Acanthamoeba* spp., are widely distributed in soil, water, and air. They are capable of causing granulomatous amebic encephalitis, *Acanthamoeba* pneumonia, *Acanthamoeba* keratitis, and disseminated acanthamoebiasis. Despite low occurrence worldwide, the mortality rate of *Acanthamoeba* spp. infections is very high, especially in immunosuppressed hosts. *Acanthamoeba* infections are a medical problem, owing to limited improvement in diagnostics and treatment, which is associated with incomplete knowledge of pathophysiology, pathogenesis, and the host immune response against *Acanthamoeba* spp. infection. The aim of this review is to present the biochemical and molecular mechanisms of *Acanthamoeba* spp.–host interactions, including the expression of Toll-like receptors, mechanisms of an immune response, the activity of metalloproteinases, the secretion of antioxidant enzymes, and the expression and activity of cyclooxygenases. We show the relationship between *Acanthamoeba* spp. and the host at the cellular level and host defense reactions that lead to changes in the selected host’s organs.

## 1. Introduction

*Acanthamoeba* spp. are free-living amoebas (FLA) widely distributed in the environment, including soil, water, and air [1]. They are capable of causing cerebral (granulomatous amebic encephalitis, GAE) and extracerebral (*Acanthamoeba* keratitis, AK; *Acanthamoeba* pneumonia, AP; cutaneous acanthamoebiasis and disseminated acanthamoebiasis) life-threatening infections among humans [2]. *Acanthamoeba* spp. exist as an active (trophozoite) and dormant (cyst) forms. The cysts can abide and survive in various environments for years and transform into trophozoites under favorable environmental conditions [3].

Cerebral and extracerebral infections caused by *Acanthamoeba* spp. occur mostly among immunocompromised individuals, including HIV positive, organ transplant recipients, patients with chronic diseases, and those who undergo immunosuppressive therapy. Acanthamoebiasis has also been observed in immunocompetent individuals [4,5]. Despite low occurrence worldwide, the mortality rate of *Acanthamoeba* spp. infections is very high. The pathogenesis of acanthamoebiasis is not fully understood. *Acanthamoeba* spp. may invade through different ways, such as the respiratory tract or breaks in the skin, resulting in hematogenous dissemination to the brain [6]. *Acanthamoeba* spp. infections cases are often underdiagnosed and hence strong clinical suspicion along with laboratory technical expertise is required for early diagnosis and therapeutic intervention [7]. The review aims to present the biochemical and molecular mechanisms of *Acanthamoeba* spp.–host interactions based on the available data.

### 1.1. Biology of Acanthamoeba spp.

*Acanthamoeba* spp. exist in two distinct forms: an actively feeding and dividing trophozoite (15–45 μm) and a dormant cyst stage (12–25 μm) [8]. Both stages are infective to humans. Trophozoites of *Acanthamoeba* spp. actively feed on bacteria, yeast, algae and small organic particles. Cysts are formed after exposure to unfavorable environmental conditions, including changes in humidity, temperature, or environmental pH [4]. Cysts are resistant to many chemical and physical factors, including UV radiation, osmotic pressure, disinfectants, and antiseptics [9,10]. They can survive in the environment for more than 20 years [11].

### 1.2. Genome of Acanthamoeba spp.

*Acanthamoeba* species are identified based on the analysis of the diagnostic fragment 3 (DF3) region of the ribosomal DNA gene, designated *Acanthamoeba*-specific amplimer S1 (ASA.S1) [12]. DF3 encodes the highly variable stem 29-1 of the nuclear small subunit 18S rRNA gene [13]. *Acanthamoeba* genotypes are distinguished by a 5% or greater sequence difference between isolates [14,15]. To date, 22 sequence types have been identified, designated T1 through T22 [16]. However, Booton et al. [17], examining the DF3 subregion of ASA.S1 in the samples of T3 and T4 isolates, observed several unique sequences within T3 and T4 isolates. These sequences were designated T3/1–T3/5 and T4/1–T4/10 [17]. Due to the fact that different laboratories applied redundant numerical labels to identify different sequences, Fuerst and Booton [16] suggested new subtypes for the T4 genotype, which have been labeled T4A–T4F and T4Neff. The authors also described different sequences within T3 (labeled T3/01–T3/13), T5 (labeled T5/01–T5/15), T11 (labeled T11/01–T11/14), and T15 (labeled T15/01–T15/11). Types T2 and T6 are the most closely related pair of sequence types within *Acanthamoeba*, and these two types are considered as a supergroup in which five subtypes are formed: T2, T2/6A, T2/6B, T2/6C and T6 [16].

Among all genotypes, T4 is the most frequently isolated from nature, and it includes many pathogenic strains that have been associated with neurological and pulmonary acanthamoebiasis. It is assumed that T4 is characterized by increased virulence and increased resistance to chemotherapeutic agents [18]. The genotypes T1, T2, T4, T5, T10, T11, and T12 are the factors of granulomatous amebic encephalitis [13,19], whereas T2, T4, T5, T16, and T18 may be the factors of *Acanthamoeba* pneumonia [20,21,22,23].

### 1.3. Occurrence of Acanthamoeba spp. in the Environment

*Acanthamoeba* spp. are free-living protozoa that are widely distributed in the environment. Trophozoites and cysts of *Acanthamoeba* spp. with varying degrees of pathogenicity were found in the water, soil, and air samples. They occur in the rivers, seas, ocean sediments, lakes, ponds, hot springs, water sewage, swimming pools, rainwater, and even in mineral and bottled water [5]. In recent years, *Acanthamoeba* spp. have been isolated from tap water in Lithuania and city water in Iran [24,25]. In Poland, Derda et al. [26] isolated *Acanthamoeba* spp. from fountains, and Górnik and Kuźna-Grygiel [27] found the presence of pathogenic *Acanthamoeba* strains in natural recreational tanks and indoor as well as outdoor swimming pools. *Acanthamoeba* spp. strains were also isolated from potting soil, dust, air conditioning, dental units and dialysis stations [28]. Besides, amoebas were isolated from vegetables, fruits, mushrooms, but also from biological materials, such as swabs from the nasal mucosa, throat, and purulent secretions from the ear [26]. In Nigeria, the colonization of nasal mucosa by *Acanthamoeba* spp. was found in 24% of the studied participants [29]. The ubiquity of *Acanthamoeba* spp. is confirmed by the fact that 80% of the human population has natural IgG antibodies against *Acanthamoeba* spp. [30].

## 2. Granulomatous Amebic Encephalitis (GAE)

### 2.1. Epidemiology

*Acanthamoeba* spp. causes rare fatal cerebral infection, particularly among immunocompromised patients [31]. The mortality rate of GAE is around 97–98% [4]. In Pubmed, since 1990–2020, 75 cases of patients with GAE caused by *Acanthamoeba* spp. have been published. Most patients came from the USA, but there were also cases from India, Austria, China, Turkey, Italy, Thailand, Japan, Spain, Germany, Peru, Taiwan, Venezuela, Sweden, Canada, Saudi Arabia, Mexico, Iran and United Kingdom [5]. However, due to non-specific symptoms and diagnostic difficulties, it is presumed that many cases were not recognized or incorrectly diagnosed as bacterial, viral, or fungal infections [32,33,34]. GAE occurs mostly among immunocompromised patients, but immunocompetent cases with GAE have also been reported. Nineteen patients survived cerebral infection of *Acanthamoeba* spp., among which twelve patients (63%) were immunocompetent (Table 1). All patients received combination therapy, apart from one patient; there is no information about treatment. Risk factors associated with GAE include immunosuppressive states such as acquired immunodeficiency syndrome (AIDS), hematological malignancies, organ transplantation, intake of steroids or other immunosuppressive therapy, systemic lupus erythematosus, diabetes mellitus, and other factors such as prolonged and excessive use of antibiotics, chronic alcoholism, liver cirrhosis, malnutrition, pregnancy, surgical trauma, burns, wounds, and radiation therapy [6,35].

### 2.2. Symptoms

The nasal cavity is usually the gateway to infestation. The *Acanthamoeba* spp. infection mostly occurs through inhalation of air or aspiration of water contaminated with an infective stages of these parasites [54]. Trophozoites migrate to the central nervous system (CNS) through the nasal mucosa and the endothelium of the brain’s capillaries [55]. Oral mucosa, damaged or ulcerated skin, and intestinal mucosa can also be sites of infection [56]. It is assumed that penetration into the CNS of the hosts may occur through the bloodstream, mainly from primary lesions in the skin, lungs, or kidneys. The mechanisms involved in the pathogenesis of GAE are not fully understood. Infection is chronic or subacute, with focal necrotic lesions of the brain leading to death 8 days to several months after the onset of symptoms [56]. Clinical symptoms associated with the presence of parasites in the brainstem are as follows: diencephalon or disorientation, lethargy, changes in behavior with hallucinations and irritability, as well as disturbances in taste and smell. The patients also had a headache, especially in the frontal area, neck stiffness, changes in body temperature, seizures, epilepsy, and nausea [9,11].

### 2.3. Mechanisms Involved in GAE

#### 2.3.1. Immune Responses

The activation of a specific immune response takes time, so in the first days of the infection, non-specific response mechanisms are important as the first line of defense. Toll-like receptors (TLRs) identify highly conserved structural motifs, known as pathogen-associated microbial patterns (PAMPs), which are expressed by microorganisms, or damage-associated molecular patterns (DAMPs) that are endogenous particles released from necrotic or dying cells [57,58]. The main task of TLRs is to activate the cells of the immune system. They are found on the surface of many cells, such as dendritic cells, mast cells, monocytes, neutrophils, and macrophages. In the brain, TLR2 and TLR4 are expressed on microglia, oligodendrocytes, astrocytes, and in neurons [59,60]. The best known TLRs are TLR2 and TLR4. On the basis of experimental studies, it was noted that the ligands for TLR2 are the products of decay of dead cells [61], and the ligands for TLR4 are heat shock proteins, which *Acanthamoeba* spp. have on their surface [62].

There are far fewer papers concerning the role of TLR in response to parasitic infections. There are also few data on the role of TLR in the innate and acquired responses of the host immune system during acanthamoebiasis. Wojtkowiak-Giera et al. [63] studied the expression of TLR2 and TLR4 in the brain of uninfected and *Acanthamoeba* spp. infected mice using quantitative polymerase chain reaction (qPCR) and immunohistochemistry (IHC) methods. Using both methods, the uninfected mice showed very similar mRNA expression of TLR2 and TLR4. In the brains of *Acanthamoeba* spp. infected mice, the TLR2 expression was significantly higher than the TLR4 expression. Wojtkowiak-Giera et al. [63] reported that the TLR2 expression statistically increased at the beginning of infection (at 2 and 4 days post *Acanthamoeba* spp. infection, dpi) and then decreased (16 and 30 dpi) to the level similar to the uninfected mice. The TLR4 expression in the brains of *Acanthamoeba* spp. infected mice increased only at 2 dpi, whereas at 4, 16, and 30 dpi, it was at a similar level compared with uninfected mice. Observed changes in the TLR2 and TLR4 expressions in the brain of *Acanthamoeba* spp. suggest the role of these receptors in the recognition of *Acanthamoeba* spp. molecules and in the pathomechanism of GAE [63].

The role of TLR receptors in the activation of Th1 (cellular) and Th2 (humoral) responses, acquired immune responses, has been well described in bacterial infections [64,65]. The activation of TLR induces the expression of antigen-presenting cell (APC)-derived cytokines, such as interleukin (IL)-12, IL-23, and IL-27, which promote the differentiation of Th1 cells and inhibit the differentiation of Th2 cells, which preferentially secrete IL-4, IL-5, and IL-13 [66,67]. Relatively few studies have looked at TLR response induction in parasite infections. Parasites can both activate and inhibit the signal across TLRs [68], but the role of these mechanisms in the parasite–host interaction requires further research. Massilamany et al. [69] characterized the inflammatory cells in the brains and spinal cords of *Acanthamoeba castellanii* infected mice. The infiltrates were comprised of both T cells (~51%) and non-T cells (~49%). Non-T cells were represented by macrophages, dendritic cells, neutrophils, B cells, and natural killer cells. The proportions of CD4 T cells and CD8 T cells were similar [69]. The authors determined the frequencies of Th1, Th2, and Th17 cytokine-producing cells based on intracellular staining by flow cytometry. The analyses included a panel of cytokines: interferon gamma, IFN-γ (Th1 cells), IL-4 and IL-10 (Th2 cells), and IL-17A, IL-17F, and IL-22 (Th17 cells). It was reported that antigen-sensitized lymphocytes from *A. castellanii*-infected mice contain mostly Th1 cytokine-producing cells; the levels of Th2 and Th17 were similar. The cytokines can be arranged in the following order: IFN-γ> IL-10 > IL-17F > IL-4 or IL-17A or IL-22. The level of IFN-γ-producing cells were three-to-four times higher than Th2 or Th17 cytokine-producing cells. Massilamany et al. [69] showed that *Acanthamoeba* spp. infection can generate encephalitogenic cross-reactive T cells by antigenic mimicry, and *Acanthamoeba* antigen-specific T cells are capable of secreting IFN-γ.

#### 2.3.2. Matrix Metalloproteinases (MMPs) and Tissue Inhibitors of MMPs (TIMPs)

MMPs are proteins that play an important role in regulating processes, such as cell differentiation and migration, the regulation of growth factor activity, angiogenesis, and the development of inflammation. MMPs also influence cell survival or its entry into apoptosis, and cell-to-cell communication [70,71]. To date, more than 20 subtypes of MMPs have been identified. The activity and expression of MMPs are controlled mainly (i) by the action of tissue inhibitors of metalloproteinases—TIMPs, (ii) by proenzyme activation, because MMPs are initially synthesized in the form of inactive zymogens, (iii) and by the regulation of gene transcription [72]. Metalloproteinases can be viewed as mediators of nervous system disorders [73]. Increased levels of MMPs are observed in CNS lesions and diseases, such as stroke, multiple sclerosis, and neuropathy; the increased concentration of these enzymes is caused not only by the synthesis from incoming leukocytes, but also by increased synthesis in brain neurons and endothelial cells. In the course of CNS damage, the activity of MMPs is increased under the influence of inflammatory cytokines and chemokines, which causes the interactions between MMPs and nitric oxide (NO), reactive oxygen species (ROS), extracellular matrix (ECM), growth factors and their receptors, integrins and other proteins. The degradation of cytokines by MMPs modulates inflammation, either inducing a worsening and neurotoxic effect or extinguishing it, which in turn promotes cell survival [73,74,75].

Metalloproteinases are found in some parasitic infections of CNS, including *Plasmodium falciparum*, *Trypanosoma brucei* spp., and *Toxoplasma gondii* [76]. In *Naegleria fowleri*, which is also free-living amoeba with high neurotropism, MMP-2 was found as an integral membrane protein in trophozoites, whereas MMP-9 was either a molecule in the cytosol or a molecule in peripheral membrane. However, the presence of MMPs did not correspond with the virulence of the strain [77]. The secretion of metalloproteinases plays a role in the parasite invasion of the CNS, which elicits the thought that the inhibition of MMPs may hinder the early invasion process [76,77]. To date, it has been reported that *Acanthamoeba* spp. express and activate several virulence proteins, which allow them to penetrate the biological barriers of the host. Lorenzo-Morales et al. [8] reported that *Acanthamoeba* spp. produce contact-dependent metalloproteinases during the adhesion to host cells. These proteinases induce the degradation of membranes and host cell death [8].

Imbalances in MMPs and their TIMPs in the selected structures of immunocompetent and immunosuppressed mice were reported by Łanocha-Arendarczyk et al. [78]. The activity of MMPs (−2 and −9) and TIMPs (−1 and −3) in the hippocampus and cerebral cortex were measured by enzyme-linked immunosorbent assay (ELISA) and IHC methods. Immunocompetent *Acanthamoeba* spp.-infected mice at the beginning of infection showed higher activity of MMP-9 in the cerebral cortex compared to the uninfected group of mice, whereas immunosuppressed *Acanthamoeba* spp.-infected mice at 24 dpi showed lower MMP-9 levels compared to immunosuppressed uninfected animals. Moreover, MMP-9 level in *Acanthamoeba* spp.-infected mice differed between the hippocampus and cerebral cortex at 16 dpi. The authors also determined the activity of MMP2 in the cerebral cortex and hippocampus of immunocompetent and immunocompromised hosts, but none of the differences were reported. Łanocha-Arendarczyk et al. [78] found a higher level of TIMP-1 protein in the cerebral cortex of immunocompetent *Acanthamoeba* spp.-infected mice than in the uninfected mice at 8 dpi, and lower TIMP-3 level in the hippocampus of immunosuppressed *Acanthamoeba* spp.-infected mice compared to the immunosuppressed uninfected mice at 16 dpi. The MMP-9/TIMP-1 ratio was greater in the hippocampus of the immunosuppressed *Acanthamoeba* spp.-infected mice than of the uninfected animals at the beginning of infection. A similar trend was reported in *Acanthamoeba* spp.-infected immunosuppressed mice in an MMP-2/TIMP-3 ratio at 8 dpi, but the difference was not significant. Łanocha-Arendarczyk et al. [78] indicate that the increase in the activity of matrix metalloproteinases during acanthamoebiasis may be primarily the result of the inflammation process, probably an increased activity of proteolytic processes, but also a defense mechanism, preventing the processes of neurodegeneration. Moreover, the authors suggest that MMPs might represent suitable therapeutic targets to prevent the unsealing of the brain–blood barrier in inducted amoebic brain infection [78].

#### 2.3.3. Neurotrophins: Brain-Derived Neurotrophic Factor (BDNF) and Neutrotrophin-4 (NT-4)

Brain-derived neurotrophic factor (BDNF) and neurotrophin-4 (NT-4) are members of the neurotrophin family. BDNF supports the differentiation, maturation, and survival of neurons in the central and peripheral nervous system and shows a neuroprotective effect under adverse conditions, such as hypoglycaemia, cerebral ischemia, neurotoxicity [79]. BDNF stimulates and controls the growth of new neurons from neural stem cells (neurogenesis) [80,81]. BDNF protein and mRNA have been reported in most brain structures, including the hippocampus, cortex, olfactory bulb, and spinal cord [81]. Decreased BDNF levels were observed in neurodegenerative diseases, such as multiple sclerosis [82], Parkinson’s disease [83], and Hungtington’s disease [84]. By contrast, increased BDNF expression was reported in patients with severe cerebral malaria [85] and ocular toxoplasmosis [86]. Łanocha-Arendarczyk et al. [87] determined BDNF levels in the hippocampus and cerebral cortex of immunocompetent and immunocompromised mice infected with *Acanthamoeba* spp. using the ELISA method. The highest level of BDNF was reported in the hippocampus and cerebral cortex in the mice at the beginning of *Acanthamoeba* spp. infection (8 dpi). Increased BDNF level in the immunocompetent and immunosuppressed *Acanthamoeba* spp.-infected mice correlated with neurological symptoms of mice. Along with the duration of infection, the level of BDNF was decreasing in both immunocompetent and immunosuppressed *Acanthamoeba* spp.-infected mice. Łanocha-Arendarczyk et al. [87] reported no differences in the BDNF level between the hippocampus and cerebral cortex of *Acanthamoeba* spp.-infected mice and no differences between immunocompetent and immunosuppressed animals. On the basis of the study, it was reported that increased BDNF level in the brain may have neuroprotective effects, and the reduction in the activity of this factor may be related to the progressive process of atrophy and/or neuronal death, usually observed in the cerebral form of acanthamoebiasis [87].

Neurotrophin-4 plays an important role in the development of the nervous system. NT-4 has a similar role to BDNF; it controls the survival and differentiation of vertebrate neurons [88]. NT-4 might also play a role in long-term potentiation and plasticity [89,90]. Chan et al. [91] showed that treatment with NT-4 reduced the infarction volume in a permanent focal cerebral ischemic rat model, demonstrating that NT-4 is involved in ischemic brain injury. Chung et al. [92] reported that NT-4 might participate in the recovery process in brain damage. Łanocha-Arendarczyk et al. [93] determined the activity of NT-4 in the cerebral cortex and hippocampus of mice infected with *Acanthamoeba* spp. with regard to the immunological status of animals using ELISA method. The highest activity of NT-4 was noticed in the cerebral cortex and hippocampus of immunocompetent and immunosuppressed mice on the 8th day post *Acanthamoeba* spp. infection. In immunocompetent infected mice, NT-4 activity decreased with the duration of the infection. Łanocha-Arendarczyk et al. [93] reported the differing activity of NT-4 between the cerebral cortex and hippocampus of immunocompetent mice at 16 dpi and immunosuppressed mice at 8 dpi.

#### 2.3.4. Histopathological Changes

Based on the results of the research and the analysis of clinical cases, it was found that *Acanthamoeba* spp. exhibited the properties of a parasite with noticeable neurotropism. The first experimental studies on the pathogenicity of *Acanthamoeba* spp. were carried out by Culbertson et al. [94]. The authors found that the intranasal administration of amoebas resulted in the death of mice within 4 days of *Acanthamoeba* spp. infection. Ulceration of the nasal mucosa was observed in histological preparations, and the presence of amoebas was observed in the frontal lobes of the brain [94]. In another study, Culbertson et al. [95] observed that *Acanthamoeba* spp., by destroying the nasal mucosa and olfactory bulbs, caused fatal changes in the brain, often accompanied by changes in the lungs. In 1966, Culbertson et al. [96] isolated new strains of *Acanthamoeba* spp. that induced granulomas forming in the forebrain, mainly in the olfactory bulbs. Histological analysis showed that the occurrence of amoebas in the brain was accompanied by an infiltration of neutrophilic granulocytes, gradually changing into infiltration, consisting of mononuclear cells [96]. Extensive inflammatory lesions of the brain, including the olfactory and frontal lobes, were found in mice infected intranasally with strains of free-living amoebas isolated from lakes in Poznań, central Poland [97]. Diffuse infarctions of nerve and glial cells in the nervous tissue were found [97]. In the study of histological changes in the brain of mice infected with amoebas isolated from water reservoirs in the Lublin region (east part of Poland), Gieryng and Gieryng [98] observed general cerebral congestion with large ecchymoses in the area of the frontal and parietal lobes and the cerebellum. There were small pus spots in the superficial substance of the cerebral cortex and in the olfactory bulbs, which were usually clearly red and showed necrotic lesions. Histological and morphological analysis of the brain of mice infected intranasally with environmental *Acanthamoeba* strains from northwestern Poland revealed swelling, shallowing of the furrows, and severe congestion of the meninges [27]. Moreover, the detachment of the meninges from the cortical part of the cerebral hemispheres was observed, mainly in the area of the frontal lobes [27]. It was found that there is a close relationship between the severity of inflammation in the brain and the survival time of the mice. In animals that died in the first days post *Acanthamoeba* spp. infection, slight brain lesions were observed, while in mice that survived 10–17 days, the greatest level of brain damage was observed [98]. Similarly, Rucka [97] found that the degree of intensification of inflammatory processes in the brain depended on the survival time of mice experimentally infected with amoebic strains. It was also noted that only trophozoites of *Acanthamoeba* spp. appeared in the brain of animals infected with amoebas [98], while Martinez [99] observed both trophozoites and cysts of *Acanthamoeba* spp. in the brain of a patient with granulomatous encephalitis.

Histopathological examinations of the brains of patients with *Acanthamoeba* spp. infections showed cerebral edema and numerous necrotic as well as hemorrhagic areas. The brainstem, hemispheres, and cerebellum showed effusions and inflammatory infiltrates consisting of neutrophils, macrophages, mononuclear cells, and multinuclear giant cells. Necrotizing arteritis, selective accumulation of trophozoites and cysts of *Acanthamoeba* spp. in the perivascular spaces were also observed. The cerebrospinal fluid (CSF) examination revealed lymphocyte-predominant pleocytosis, elevated protein levels, and normal or low glucose levels [2,100,101].

**Figure 1:**
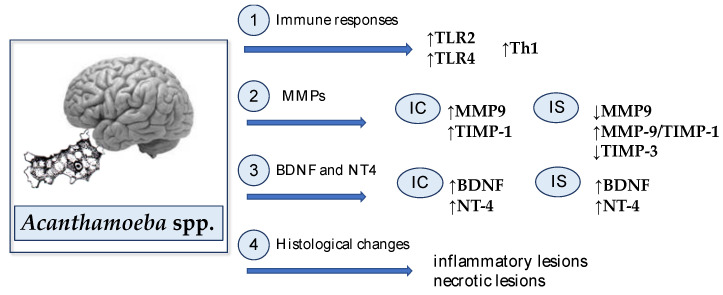
Host defense mechanisms against *Acanthamoeba* spp. infection in the brain: 1. *Acanthamoeba* spp. infection affects the growth of Toll-like receptors (TLR) expression, mainly TLR2 and TLR4. Moreover, it was found that antigen-sensitized lymphocytes from the brain of *Acanthamoeba* spp. infected mice contain mostly Th1 cytokine-producing cells; 2. Acanthamoebiasis causes an increase in the activity of metalloproteinases-9 (MMP-9) and the tissue inhibitor of metalloproteinases-1 (TIMP-1) in immunocompetent hosts (IC), and a decrease in MMP-9 and TIMP-3 in immunosuppressed hosts (IS). In immunocompromised hosts, an increase in the ratio of MMP-9/ TIMP-1 was also observed; 3. *Acanthamoeba* spp. infection affects the growth of brain derived neurotrophic factor (BDNF) and neurotrophin-4 (NT-4) activity in both immunocompetent (IC) and immunosuppressed (IS) hosts; 4. The amoebas induced inflammatory and necrotic lesions in the brain structures.

All described mechanisms in the *Acanthamoeba* spp. infected brain are presented in Figure 1.

## 3. *Acanthamoeba* pneumonia (AP)

### 3.1. Epidemiology

So far, 19 case reports of *Acanthamoeba* pneumonia (AP) or disseminated acanthamoebiasis with lung infection have been published. Most patients came from the USA, but there were also cases from Poland, Austria, France, Korea, Japan, and India (Table 2). None of the patients survived.

### 3.2. Symptoms

*Acanthamoeba* spp. infection to the lungs occurs mostly in patients with a low immune response [102,104,112]. The pathomechanism of AP is not clear. *Acanthamoeba* pneumonia was found in patients not only as a site of *Acanthamoeba* spp. infection [104,112] but also as a disseminated infection [102]. In patients with AP, a decrease in body weight and respiratory efficiency was observed, and in the radiological examination, interstitial changes with visible pulmonary edema were observed [112]. Martinez [105] found multiple nodular lesions in the patient’s lungs infected with *A. castellanii*. Additionally, it has been noticed that the infection of amoebas into the lungs may be bilateral with patchy infiltrates [102]. Im and Kim [112] noticed changes in peripheral blood parameters in a 7-month-old AP patient. During the infection, there was a decreasing trend in the level of hemoglobin (15.0–11.1 mg/dl), hematocrit (48.0–37.6%), and a decrease in the number of white blood cells (10,900–5460/mm^3^) and platelets (253–65/10^3^). However, no changes were found in the sodium and chloride levels [112]. Infection is most often diagnosed post mortem by isolating amoebas from bronchoaspirate fluid, bronchoalveolar lavage (BAL), bronchial washing, and a fragment of lung tissue obtained during a biopsy [20,102,103,104,114].

### 3.3. Mechanisms Involved in AP

#### 3.3.1. Immune Responses

TLRs are components of host defense activation in the case of infectious and non-infectious pulmonary disorders, such as acute lung injury, interstitial lung diseases, asthma, and chronic obstructive pulmonary disease. Receptors also play an important role in lung cancer [115,116]. Derda et al. [117] determined the TLR2 and TLR4 expressions in the lungs of *Acanthamoeba* spp.-infected mice using qPCR and IHC methods. The authors found that the TLR2 expression was higher in mice at 2, 4, 16, and 30 days post *Acanthamoeba* spp. infection than the TLR4 expression. The authors reported an increased expression of both receptors from 2 to 30 dpi, compared with the uninfected mice. Statistically significant differences between the uninfected and *Acanthamoeba* spp. infected mice were observed in TLR2 and TLR4 expressions at 16 dpi [117]. Derda et al. [117] showed that TLR2 and TLR4 were upregulated in the lungs of *Acanthamoeba* spp. infected mice and TLRs were located in the interstitial cells, pneumocytes, and the apical portion of epithelial cells of the bronchial tree. The authors suggested that TLRs are involved in the recognition of *Acanthamoeba* spp. in the lungs.

PAMPs are recognized by a diverse set of pattern recognition receptors (PRRs), such as Toll-like receptors, C-type lectin receptors, and protease-activated receptor (PAR) 2. The immune response may be polarized towards Th1, Th2, Th17, or Treg lymphocytes. Mechanisms of inducing Th-1 mediated by TLRs are well understood [118], whereas the mechanisms triggering other types of responses are not clear. In the case of a Th2-mediated response, there are two hypotheses concerning the mechanisms that trigger this response: (i) it develops in the absence or incomplete induction of Th1 inflammation, or (ii) there are distinct receptors and signaling pathways responsible for recognizing PAMP pathogens, including the Th2 response [119].

An airway allergic reaction and the presence of parasites in the host organism often causes an increased synthesis of immunoglobulin E (IgE) and Th2 interleukins, such as IL-4, IL-5, and IL-13. These cytokines lead to the recruitment of basophils, eosinophils, and mast cells. As a consequence, this mechanism causes damage to the parasite and its expulsion from the host organism [120,121]. Park et al. [122] determined the activity of several chemokines in the bronchoalveolar lavage fluid (BALF) of mice infected with low (100 trophozoites) and high dose (4 × 10^5^ trophozoites) of *A. lugdunensis*. In mice infected with a high dose of trophozoites—the levels of IL-4, IL-5, and IL-13 in BALF increased compared to uninfected animals. The IL-17 levels in BALF of *A. lugdunensis*-infected mice did not change. It was found that *A. lugdunensis* excretory–secretory (ES) proteins induced the activation of dendric cells (DCs), which accelerated the differentiation of naive CD4+ T cells into Th2 cells [122]. The authors also determine whether PAR2 mediates the airway inflammation induced by proteins that are expressed on *Acanthamoeba* spp. trophozoites. PAR2 knockout and wild-type mice were inoculated by *Acanthamoeba* ES proteins. The number of eosinophils and neutrophils was higher in the ES protein administered hosts than in uninfected mice. The lungs of PAR2 knockout and wild-type mice-treated ES proteins showed the hyperplasia of lung epithelial cells, enhanced mucin production, and immune cell infiltration around the bronchial tracts. The cytokine levels, such as IL-4, IL-5, and IL-13, increased in BALF of mice; nonetheless, the levels of studied cytokines were lower in the PAR2 knockout mice. Park et al. [122] suggest that *Acanthamoeba* trophozoites promoted Th2 responses by activating DCs via PAR2 signaling in the lungs of hosts.

#### 3.3.2. Cyclooxygenases (COXs)

The inflammatory process in the lungs increases the expression of many inflammatory proteins, including cytokines, chemokines, and adhesion molecules. It can also change the activity of enzymes related to the synthesis of eicosanoids, which play the role of the most peripherally located secondary informants in the cell [123,124]. These compounds locally in the cell affect the activity of hormones and neurotransmitters, and the profile of eicosanoids formed depends on the enzymatic composition of individual cells. Cyclooxygenases (COX) are responsible for the formation of prostaglandins, including strongly pro-inflammatory PGE2 and thromboxane (TXB2), which is an activator of platelet aggregation, a factor promoting vasoconstriction, and a strong mitogen of smooth muscles [125,126]. Cyclooxygenase, also known as prostaglandin H synthase, is an enzyme that catalyzes the conversion of arachidonic acid to prostaglandins (PGs), prostacyclins (PGIs), and thromboxanes (TXBs). In the human body, it exists in the form of two isoforms—cyclooxygenase-1 (COX-1) and cyclooxygenase-2 (COX-2) [124]. COX-1 is constantly expressed in almost all tissues and is responsible for maintaining the homeostasis of the organism, e.g., by the cytoprotection of mucous membranes, platelet aggregation, and the maintenance of normal renal blood flow. The presence of COX-1 has been demonstrated in both the upper and lower respiratory tracts, as well as in the pleura [127,128]. COX-2 is an inducible enzyme that is active in cells involved in pathological processes, such as the onset and maintenance of acute and chronic inflammation, pain, fever, and cancer. COX-2 expression has been reported to be associated with inflammation or proliferative changes in the airway epithelium, including pneumonia and lung cancer [129]. COX-2 is induced by parasitic infections, but the mechanism is not clear [130].

An experimental study shows that COX-1 and COX-2 are important mediators of the AP [131]. Łanocha-Arendarczyk et al. [131] aimed to check the impact of *Acanthamoeba* spp. infection on the transformation of arachidonic acid and the production of eicosanoids (PGs and TXs) in the lungs of immunocompetent and immunosuppressed mice using IHC and Western blot methods. Molecular analysis showed that the strong expression of COX-1 and COX-2 in the lungs of the immunocompetent host induced by *Acanthamoeba* spp. did not correspond to the significant differences in the levels of PGE2 and TXB2. It is likely that the inflammatory process caused by the parasite initiated the immune response, consisting of the inhibition of the enzyme activity by its product. The highest expressions of COX-1 and COX-2 proteins in the lungs were observed in 24 days post *Acanthamoeba* spp.-infected immunocompetent mice, and they were, respectively, 44% and 80% higher than in the uninfected animals. In contrast, steroid-induced immunosuppression in *Acanthamoeba* spp.-infected mice reduced the COX-1 and COX-2 expressions in lung, but not at the beginning of the infection. Moreover, the analysis of IHC showed a decrease in the detection of COX-1 in the *Acanthamoeba* spp.-infected immunosuppressed mice, compared to the *Acanthamoeba* spp.-infected immunocompetent mice. Łanocha-Arendarczyk et al. [131] concluded that *Acanthamoeba* spp. increase the expression and activity of COX-1 and COX-2 in the lungs of the hosts. Moreover, the authors [131] suggested that increased expression may be a result of a direct regulatory function of pulmonary epithelial cells and the release of pro-inflammatory cytokines from alveolar macrophages, or it may be related to increased levels of free radicals and decreased antioxidant enzyme activity in pulmonary acanthamoebiasis.

#### 3.3.3. Antioxidant Defense

An intense or long-lasting state of oxidative stress may induce pathological reactions, leading to cell and tissue damage. The biological consequences of the pro and antioxidant imbalance include protein denaturation, lipid peroxidation, carbohydrate damage, changes in DNA structure leading to mutations, or cytotoxic effects [132]. Increasing oxidative damage in cells is the molecular basis of the development of many diseases, also in the inflammatory processes of the lungs [133]. The violation of the oxidation-reduction potential balance also plays a role in the pathogenesis of parasitic diseases.

The parasite–host interactions are influenced by the defense mechanisms of the parasite and the host. Parasites have developed mechanisms to avoid the effects of ROS. Protozoa and helminths have at least one of the three main antioxidant enzymes that play a key role in defense response against the host’s reaction [134]. Two forms of superoxide dismutases (Fe-SOD and Cu, Zn-SOD) have been detected in *Acanthamoeba* spp. [8]. Dismutases in *Acanthamoeba* spp. may not only protect them from oxidative stress but also protect against ROS released by the cells of the immune system upon contact with amoebas [135,136]. Moreover, a correlation between the activity of glutathione peroxidase (GPx) in *Acanthamoeba* spp. and the invasiveness of these amoebas was established. The role of catalase (CAT) in the antioxidant defense system of *Acanthamoeba* spp. was not found [137]. Despite the presence of two antioxidant enzymes (SOD and GPx) in *Acanthamoeba* spp., it has been found that the increased production of ROS inhibits development and, consequently, degrades the amoeba cells [138].

Łanocha-Arendarczyk et al. [139] assessed the severity of oxidative stress by measuring the concentration of lipid peroxidation products in the lungs of mice infected with the *Acanthamoeba* AM 22 strain and assessed the effectiveness of protection against oxidative stress by measuring the activity of antioxidant enzymes (SOD, CAT, glutathione reductase (GR), GPx) regarding the immune status of the host. The study showed a significant increase in lipid peroxidation products (LPO) in immunocompetent and immunocompromised mice at 8- and 16-days post *Acanthamoeba* spp. infection, compared to uninfected mice. It was also reported that, at the beginning of the infection, the LPO levels were significantly different between immunocompetent and immunocompromised mice infected with *Acanthamoeba* spp. A significant increase in LPO was demonstrated in *Acanthamoeba* spp. infected immunocompetent mice compared to *Acanthamoeba* spp. infected immunocompromised mice. The study has shown that the *Acanthamoeba* spp. infection of mice is accompanied by changes in the activity of antioxidant enzymes in the lungs during the course of the infection. In immunocompromised *Acanthamoeba* spp.-infected mice, significantly lower activity of SOD in the lungs was found compared to the uninfected immunocompromised mice at 8 dpi. In the lungs of immunocompetent hosts, a significant increase in CAT activity was noted at 16 days post *Acanthmoeba* spp. infection. It was shown that the activity of this enzyme was significantly higher in immunocompetent *Acanthamoeba* spp.-infected mice than in immunocompetent hosts at 16 dpi. The study also reported a decrease in GR activity in the lungs of immunosuppressed *Acanthamoeba* spp.-infected vs. uninfected mice at 24 dpi. At 8 dpi, in the lungs of immunocompetent and immunocompromised mice, a decrease in GPx activity was found compared to the control groups, as well as a significantly lower activity of this enzyme was noted in the immunocompromised mice compared to the control group in 16 dpi, which could result from the depletion of the body’s antioxidant abilities during an intensified inflammatory process taking place in the lung tissue [139].

The increased activity of the immune system during *Acanthamoeba* spp. infection may lead to a shift of the antioxidant balance in the body towards oxidation, which may initiate or aggravate inflammation. An effect of the inflammatory response in immunocompetent and immunocompromised hosts in the course of the experimental *Acanthamoeba* spp. infection is a reduction in the antioxidant potential of the lungs, resulting from changes in the activity of antioxidant enzymes. In pulmonary acanthamoebiasis, the changes in the activity of antioxidant enzymes may be a consequence of oxidative stress, which is manifested by an increase in the concentration of lipid peroxidation products [139].

#### 3.3.4. Histopathological Changes

In animal studies, Culbertson et al. [94] observed *Acanthamoeba* spp. accompanied by a strong fibrin-purulent exudate reaction in the lungs of mice after the intranasal administration of amoebas. Hemorrhage was also commonly observed. The changes also included pulmonary veins with numerous clots and embolisms. In other studies, Culbertson et al. [95] observed that low doses of amoebas caused animals death due to lung lesions. Rucka [97] found the infiltration of plasma and mononuclear cells, with the destruction of the vessel wall and bronchus, in the perivascular and peribronchial tissue in the histological examination of the lungs of mice inoculated with free-living amoebas. Large exudative cells and phlegmon-containing amoebas were present in the lung tissue. Moreover, numerous foci of necrosis and purulent inflammation were observed in the lung parenchyma [97]. Gieryng et al. [140] found the destruction of the bronchial epithelium, bronchioles, and endothelium in blood vessels, to a large extent in the histological examination of the lungs of experimentally *Acanthamoeba* spp.-infected mice. The presence of *Acanthamoeba* spp. and small hemorrhages were observed at the sites of vascular and bronchial damage. The walls of the alveoli became thickened, and in some places, the destruction of the walls by *Acanthamoeba* spp. was present. As the disease progressed, pathological changes in the lungs worsened [140]. Górnik and Kuźna-Grygiel [27] noticed the hyperplasia of the bronchiolar epithelium, thickening, and congestion of the alveolar walls in the lung of *Acanthamoeba* spp.-infected mice. Park et al. [122] reported massive inflammatory cell infiltration, bronchial epithelial cell hyperplasia, and goblet cell hyperplasia in the lungs of mice infected with a high dose of *Acanthamoeba* spp. (4 × 10^5^ trophozoites). In the lungs infected with a low dose of *Acanthamoeba* spp. (100 trophozoites), Park et al. [122] observed mild infiltration of inflammation in the lungs and the hyperplasia of some bronchial epithelial cells. Some forms of *Acanthamoeba* spp. were observed in alveoli, and several eosinophils were noticed around amoebas.

**Figure 2:**
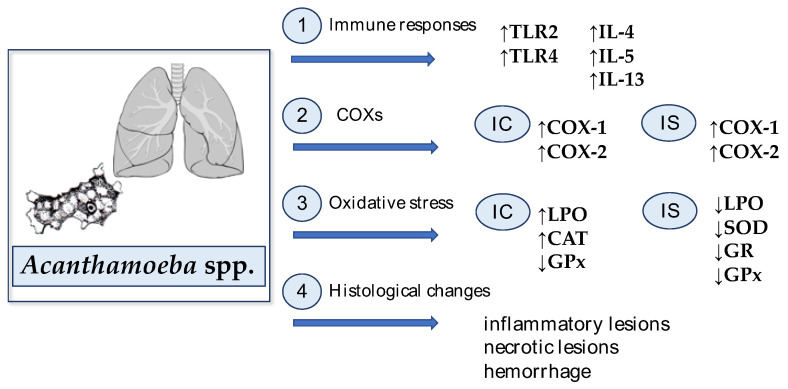
Host defense mechanisms against *Acanthamoeba* spp. infection in the lungs: 1. *Acanthamoeba* spp. infection affects the growth of Toll-like receptor (TLR) expression, mainly TLR2 and TLR4. Moreover, it was found that *Acanthamoeba* spp. increased the synthesis of Th2 cytokines, such as interleukin (IL)-4, IL-5, and IL-13; 2. Acanthamoebiasis causes an increase in expression of cyclooxygenase-1 (COX-1) and -2 (COX-2) in immunocompetent (IC) and immunosuppressed (IS) hosts; 3. *Acanthamoeba* spp. infection causes oxidative stress manifested by an increase in lipid peroxidation (LPO) and changes in antioxidant enzyme activity in the immunocompetent hosts; an increase in catalase (CAT) and a decrease in glutathione peroxidase (GPx). In immunosuppressed hosts, *Acanthamoeba* spp. causes a decrease in LPO and a decrease in superoxide dismutase (SOD), glutathione reductase (GR) and GPx; 4. The amoebas induced hemorrhage, inflammatory and necrotic lesions in the lungs.

All described mechanisms in the *Acanthamoeba* spp.-infected lungs are presented in Figure 2.

## 4. Treatment

Currently, there is no reliably effective drug therapy for *Acanthamoeba* spp. infections of the brain and lungs. Most published cases have used multiple drug combinations to provide synergistic effects and improved treatment results. Clinical cases reporting successful treatment outcomes remain limited because of the inability of most drugs to overcome the biological barriers and to penetrate into the parenchyma of the brain or lungs with sufficient concentrations to kill the amoeba. The FDA-approved drugs used in *Acanthamoeba* spp. infections are amphotericin B, azithromycin, paromomycin, itraconazole, fluconazole, ketoconazole, pentamidine, sulfadiazine, cotrimoxazole, 5-flucytosine, rifampicin, and pyrimethamine [141].

The Infectious Diseases Society of America (IDSA) guidelines for *Acanthamoeba* CNS infection have a recommendation for either fluconazole+ sulfadiazine+ pyrimethamine or trimethoprim-sulfamethoxazole+ rifampicin+ ketoconazole [142]. The Centers for Disease Control and Prevention (CDC) announced the availability of anticancer and anti-leishmaniasis drug miltefosine as a drug to treat CNS infection [143]. Table 1 presents treatment that was given to survivors of GAE; miltefosine as a part of combination therapy was used in six cases. Webster et al. [52] found that miltefosine and oral voriconazole reduced serological titers and brain lesions in immunocompetent patients with GAE. In AP, amphotericin B in combination with trimethoprim, miltefosine, voriconazole, pentamidine, 5-fluorocytosine, and itraconazole were used [21,22,102]. It is important to point out that patients with pulmonary acanthamoebiasis often receive therapy that is targeted on viral, bacterial, and fungal infections instead of *Acanthamoeba* spp. infection.

Among the potential drugs that may have possible effects on *Acanthamoeba* spp. infection, Elsheikha et al. [141] presented and described amlodipine, loperamide, prochlorperazine, a combination of chlorpromazine and rokitamycin, procyclidine, digoxin, corifungin, tigecycline, heterocyclic alkylphosphocholines (structural analogue of miltefosine) and chloroquine.

## 5. Conclusions and Future Research Perspectives

The biochemical and molecular mechanisms known to date that are involved in the response to *Acanthamoeba* spp. infection in the hosts provide valuable information on the pathogenesis and factors that affect the course of cerebral and pulmonary acanthamoebiasis. The presented mechanisms, to a small extent, allow us to understand the interaction between *Acanthamoeba* spp. and the hosts. The parasite caused the inflammation in the organs through the host’s immunological reactions, changes in antioxidant defense, metalloproteinases and cyclooxygenases activity. Owing to the fact that *Acanthamoeba* spp. are involved in a variety of processes in the host’s organism, it can be concluded that the parasite may also affect other mechanisms than those presented in this review. In an attempt to understand all the mechanisms in acanthamoebiasis, future research is needed to describe the molecular pathways of the immune response. Understanding the mechanisms of acanthamoebiasis pathogenesis may contribute to developing an effective pharmacological therapy for these infections and to limit the degree of cell damage.

## Figures and Tables

**Table 1:** Cases of survivors of granulomatous amebic encephalitis (GAE) caused by *Acanthamoeba* spp. reported during 1990–2020.

No.	Place of Study	Patient Details	Biological Sample	Treatment	References
1	India	22 years; IC	CSF	fluconazole, trimethoprim-sulfamethoxazole, metronidazole, rifampicin, miltefosin; for 3 months	[36]
2	India	3 years, male; child on chronic malnutrition	CSF	-	[13]
3	India	30 years, male; IC	CSF	rifampicin, trimethoprim-sulfamethoxazole, fluconazole; for 14 days	[37]
4	India	3 years, male; malnourished child	CSF	cotrimoxazole, rifampicin, ketoconazole	[38]
5	India	25 years, male; IC	frontotemporal craniotomy	rifampicin, trimethoprim-sulfamethoxazole, fluconazole	[39]
6	India	2 years, male; IC	CSF	vancomycin, ceftriaxone, dexamethasone	[40]
7	India	45 years, female; IC	CSF	rifampicin, cotrimoxazole, fluconazole, albendazole, carbamazepine	[41]
8	India	15 years, female; IC	CSF	ketoconazole, rifampicin, cotrimoxazole; for 9 months	[42]
9	Malaysia	1 year, female; IC	brain biopsy	fluconazole, trimethoprim, rifampicin; for 106 days	[43]
10	Spain	33 years, male; patient with AIDS	brain biopsy	fluconazole, sulfadiazine and surgical excision of lesions	[44]
11	Germany	64 years, female; IC	CSF	fluconazole, rifampicin, metronidazole, sulfadiazine; for 14 days	[45]
12	UK	41 years, male; liver transplant recipient	brain tissue	rifampicin, co-trimoxazole; for 3 months	[46]
13	Austria	25 months, male; patients with ALL	CSF	trimethoprim-sulfamethoxazole, fluconazole, pentamidine, miltefosine	[47]
14	Austria	17 years, male; IC	CSF	fluconazole, rifampicin, cotrimoxazole; for 2 months	[48]
15	Austria	25 years, male; patients with tuberculous meningitis	CSF, biopsy	fluconazole, trimethoprim-sulfamethoxazole, amphotericin B, flucytosine, sulfadiazine, miltefosine	[49]
16	Italy	35 years, male; IC	brain tissue biopsy	fluconazole, trimethoprim-sulfamethoxazole, miltefosine	[50]
17	USA	34 years, male; patient with HIV	CSF	trimethoprim-sulfamethoxazole, flucytosine, fluconazole, miltefosine	[51]
18	Canada	38 years, male; IC	serum sample, brain biopsy	voriconazole, miltefosine	[52]
19	Taiwan	63 years, male; IC	CSF	amphotericin B, rifampicin, dexamethasone	[53]

ALL, acute lymphoblastic leukemia; CSF, cerebrospinal fluid; HIV, human immunodeficiency virus; IC, immunocompetent.

**Table 2:** Cases of *Acanthamoeba* pneumonia (AP) or disseminated acanthamoebiasis with lung infection reported during 1990–2020.

No.	Place of Study	Patient Details	Biological Sample	References
1	Poland	15 years, male; patient with AML	bronchoaspirate fluid	[20]
2	Poland	53 years, male; patient with ACL	bronchoaspirate fluid	[20]
3	Poland	newborn with atypical pneumonia	bronchoalveolar lavage (BAL)	[20]
4	Poland	newborn with atypical pneumonia	bronchoalveolar lavage (BAL)	[20]
5	Austria	25 years, male from India; IS	lung biopsy	[21]
6	France	39 years, male; heart transplant recipient	postmortem, biopsy of lungs	[22]
7	USA	53 years, male; patient with ALL	postmortem, biopsy of lungs	[102]
8	USA	63 years, male; liver transplant recipient	postmortem, biopsy of lungs	[103]
9	USA	38 years, male; kidney transplant recipient	postmortem, biopsy of lungs	[104]
10	USA	28 years, male; kidney transplant recipient	postmortem, biopsy of lungs	[105]
11	USA	39 years, female; patient with CML	postmortem, biopsy of lungs	[106]
12	USA	45 years, male; patient with AML	postmortem, biopsy of lungs	[107]
13	USA	35 years, male; patient with AIDS	bronchial washing	[108]
14	USA	60 years, male; bilateral lung transplant recipient	postmortem, biopsy of lungs	[109]
15	USA	62 years, male; single lung transplant recipient	postmortem, biopsy of lungs	[110]
16	USA	60 years, male; lung transplant recipient	postmortem, biopsy of lungs	[111]
17	Korea	7 months, female; congenital immunodeficiency patient	bronchial washing	[112]
18	Japan	52 years, male; allogenic bone marrow transplantation	postmortem, biopsy of lungs	[23]
19	India	36 years, female; kidney transplant recipient	postmortem, biopsy of lungs	[113]

ACL, acute chronic marrow leukemia; AML, acute myeloblastic leukemia; ALL, acute lymphoblastic leukemia; CML, chronic myelogenous leukemia; IC, immunocompetent; IS, immunosuppressed.

## Data Availability

No new data were created or analyzed in this study. Data sharing is not applicable to this article.
